# A qualitative study describing nursing home nurses sensemaking to detect medication order discrepancies

**DOI:** 10.1186/s12913-017-2495-6

**Published:** 2017-08-04

**Authors:** Amy Vogelsmeier, Ruth A. Anderson, Allison Anbari, Lawrence Ganong, Amany Farag, MaryAnn Niemeyer

**Affiliations:** 1 S314 Sinclair School of Nursing, Columbia, MO 65211 USA; 2 University of North Carolina at Chapel Hill, 2007 Carrington Hall, Chapel Hill, NC 27599-7460 USA; 3 Missouri State University, Professional Building 305, 901 S. National Ave, Springfield, MO 65897 USA; 4 University of Missouri Department of Human Development and Family Science, 405 Gentry Hall, Columbia, MO 65211 USA; 5 University of Iowa College of Nursing, 101 College of Nursing Building, 50 Newton Road, Iowa City, IA 52242-1121 USA; 6 Sinclair School of Nursing, Columbia, MO 65211 USA

**Keywords:** Nursing homes, Sensemaking, Medication reconciliation, Medication safety

## Abstract

**Background:**

Medication reconciliation is a safety practice to identify medication order discrepancies when patients’ transitions between settings. In nursing homes, registered nurses (RNs) and licensed practical nurses (LPNs), each group with different education preparation and scope of practice responsibilities, perform medication reconciliation. However, little is known about how they differ in practice when making sense of medication orders to detect discrepancies. Therefore, the purpose of this study was to describe differences in RN and LPN sensemaking when detecting discrepancies.

**Method:**

We used a qualitative methodology in a study of 13 RNs and 13 LPNs working in 12 Midwestern United States nursing homes. We used both conventional content analysis and directed content analysis methods to analyze semi-structured interviews. Four resident transfer vignettes embedded with medication order discrepancies guided the interviews. Participants were asked to describe their roles with medication reconciliation and their rationale for identifying medication order discrepancies within the vignettes as well as to share their experiences of performing medication reconciliation. The analysis approach was guided by Weick’s Sensemaking theory.

**Results:**

RNs provided explicit stories of identifying medication order discrepancies as well as examples of clinical reasoning to assure medication order appropriateness whereas LPNs described comparing medication lists. RNs and LPNs both acknowledged competing demands, but when performing medication reconciliation, RNs were more concerned about accuracy and safety, whereas LPNs were more concerned about time.

**Conclusions:**

Nursing home nurses, particularly RNs, are in an important position to identify discrepancies that could cause resident harm. Both RNs and LPNs are valuable assets to nursing home care and keeping residents safe, yet RNs offer a unique contribution to complex processes such as medication reconciliation. Nursing home leaders must acknowledge the differences in RN and LPN contributions and make certain nurses in the most qualified role are assigned to ensure residents remain safe.

## Background

Despite efforts to improve resident safety for the 1.6 million persons residing in United States nursing homes, adverse events still occur. According to a report by the Office of the Inspector General, approximately 22% of a sample of skilled nursing facility (SNF) residents experienced at least one adverse event during their SNF stay. Fifty-nine percent of adverse events, including those related to medication use, were deemed preventable [1].

Adverse events associated with medications pose a significant risk to frail nursing home residents [2]. Preventable adverse drug events (ADEs) may result from medication order discrepancies such as drug omissions, drug additions, and dosage changes that unintentionally occur during transitions in care [3–5]. Evidence suggests that three out of four residents will have at least one medication order discrepancy at transfer to the nursing home [6]. Medication order discrepancies, defined as differences between medications a person is taking and what they are intended to be taking, can include such high-risk drugs as cardiovascular agents, opioid analgesics, neuropsychiatric agents, hypoglycemic agents, antibiotics, and anticoagulants [4, 5].

Medication reconciliation is a process designed to detect and resolve medication order discrepancies as patients’ transition between settings. According to The Joint Commission [7], medication reconciliation should involve comparing medications a person is taking or intended to be taking with newly ordered medications, looking for duplications, omissions, and interactions, and identifying if current medications are appropriate to continue. Joint Commission recommendations state that because of the complex nature of conducting medication reconciliation, it should be performed by qualified individuals identified by the organization. Experts agree that medication reconciliation should be a shared interdisciplinary process [7, 8] and in hospitals responsible individuals include physicians, pharmacists, and registered nurses (RN) [9, 10]; however, in nursing homes, licensed practical/vocational nurses (LPNs) also assume responsibility for the process [11, 12].

In an observational study of medication reconciliation in nursing homes, Vogelsmeier and colleagues [11] found that RNs and LPNs were both responsible to detect and communicate medication order discrepancies to physicians when residents transition to the nursing home. However, how nurses interpreted or made sense of medication orders to detect discrepancies differed, leading to inconsistent actions to resolve discrepancies. For example, some nurses, more often RNs, sought clarification about possible discrepancies, asking questions of others, whereas others, more often LPNs, worked in isolation, making assumptions about the presence or absence of discrepancies. However because the study involved individual nurse encounters with real time resident transfers, direct comparisons could not be made. Therefore, to better understand sensemaking differences between nursing home RNs and LPNs, this study was conducted in which standardized resident transfer vignettes were presented to participants to allow for direct comparisons. The purpose of this article is to present qualitative findings from RN and LPNs interviews to directly compare how their sensemaking differed. By understanding RN and LPN differences in sensemaking, we can begin to develop medication reconciliation tools and processes for use in the nursing home that account for these differences.

### Guiding theory

This study was guided by sensemaking theory. Weick [13] defines sensemaking as a cognitive process in which individuals construct mental models that they in turn use to interpret and assign meaning to unexpected events (e.g., adverse events). Greca and Moriera [14] suggest mental models represent situations and events, and that through mental manipulation, individuals are then capable of understanding and explaining phenomena, and act accordingly. Mental models are constructed through personal experiences and can be expanded and enhanced by education and new experiences as new information is gained. As one’s mental model is enhanced, so is their ability to effectively interpret and assign meaning to an event [14, 15].

Thus in our study of RNs and LPNs in nursing homes we expect sensemaking to differ because each group likely uses cognitive processes and mental models expanded through their different education paths and their differing scopes of practice. LPN education and subsequent roles are focused more on basic nursing care including taking vital signs, and ensuring assistance with bathing and dressing, and medication administration; any observations about change in resident status must be reported to the RN for full assessment. RN education and subsequent roles focus on comprehensive assessment, analyses of data, and care planning and oversight of LPNs and unlicensed staff [16].

Weick’s theory [13] outlines seven properties of sensemaking which he states distinguish it from other cognitive processes to facilitate understanding of how meaning is assigned. In Table 1, we define the seven properties of sensemaking and describe how RN and LPN sensemaking differs based on findings from this study. Because medication reconciliation is a complex cognitive process, analyzing our study findings through this theory helped us to better understand how nurses interpret and assign meaning to the medication order discrepancies they encounter.

**Table 1:** Sensemaking Properties: Differences Between Nursing Home RNs and LPNs

Sensemaking Properties	Explanation	Differences between Nursing Home RN and LPN Sensemaking
Identity construction	Members of organizations come to know themselves through interactions with others in the organization	RNs and LPNs perceive themselves as equal in skills and abilities despite differences in their education and licensure. This perceived equivalence poses a risk that collaboration between the roles may not routinely occur when performing medication reconciliation. LPNs also perceive themselves superior to RNs because RNs ask questions. The perception that asking questions is a sign of incompetence suggests that LPNs are less likely to ask questions when clinically necessary.
Retrospective	Individuals make sense of events after they occur	RNs past experience with identifying discrepancies provides opportunities to learn from these experiences, thus influencing the detection of future discrepancies. In contrast, because LPNs are not recognizing discrepancies, they have limited opportunity to learn from past experiences, therefore future discrepancies may go undetected and unresolved.
Enactment	Individuals in part create the environments they encounter; they mentally create what they expect to find	RNs anticipation that medication order changes will occur leads them to clarify intentional changes versus errors. LPNs on the other hand anticipation that orders are written as intended leads them to make assumptions about any changes including unintentional changes that may have occurred, thus potentially harmful errors can go undetected and unresolved.
Social	Individuals are influenced by relationships and interactions with others	Either RN or LPN likelihood of detecting discrepancies may be expanded or limited based on their interaction with either valued or adversarial colleagues.
Ongoing	An event’s history and context influence ongoing understanding of future events	RNs consider medication reconciliation within the context of resident safety and medication appropriateness, therefore placing an emphasis on medications type and the resident’s clinical history, as well as any recent events (i.e., hospitalization). LPNs on the other hand consider medication reconciliation within the context of the transfer routine and what they anticipate to occur.
Extracted cues	Individuals will extract cues out of familiar structures or known points of reference within their environment	RNs have a richer mental model than LPNs because of their expanded education; therefore RNs recognize cues differently than LPNs. Also, when nurses’ (either RN or LPN) have an overreliance on organizational cues (i.e., rules/regulations/policies) this may limit their ability to think beyond defined cues and not notice other sources of error.
Plausibility rather than accuracy	Individuals seek to behave in a reasonable fashion within the context of the unexpected event so they can move quickly past it.	Both RNs and LPNs likelihood of detecting discrepancies may be influenced by time constraints or shift pressures that cause them to overlook subtle cues or signs. However, RNs recognize the risk to safety, therefore focus more on the cognitive nature of the process in spite of time constraints.

## Methods

We conducted a qualitative study between 2013 and 2015 using both conventional and directed content analysis [17] to describe RN and LPN sensemaking when encountering medication order discrepancies at resident transitions into the nursing home. Participants, including nursing home RNs and LPNs, were asked to read four resident transfer vignettes and then respond to face-to-face interview questions about whether or not they perceived medication order discrepancies were present within the vignette. Each vignette was comprised of three parts: 1) brief description of an individual’s medical history and medication order list prior to hospitalization, 2) brief description of the individual’s acute care stay and list of medication orders at time of discharge, and 3) list of transfer orders for medications to be continued in the nursing home. Two medication order discrepancies (medication order difference between settings) were embedded in each vignette. A sample vignette and description of vignette development was previously published [18].

### Ethics and consent

The study was approved by the University of Missouri Health Sciences Institutional Review Board. Verbal permission to conduct the study was obtained by the nursing home administrators and directors of nursing. A waiver of documentation of consent was obtained from RN and LPN participants because the level of risk to participants was low. Participation was voluntary for the nursing home sites as well as participants from each site. Participants did not have a relationship with the PI or any member of the research team prior to the study.

### Setting, nurse sample, and recruitment

Twenty nursing homes were randomly selected from a sampling pool of 100 eligible nursing homes located in Midwestern United States. Eligible nursing homes, identified online through the Medicare sponsored website, Nursing Home Compare, had to be within 150 mile radius of the University of Missouri which served as the project site, 60 beds or greater in size, not located within a hospital, and participate in the Medicare and Medicaid program. RNs and LPNs from each nursing home site were eligible to participate if they worked at least 8 h per week in that nursing home and had experience conducting medication reconciliations as part of one or more resident admissions. The unit of analysis was the individual nurse, thus we did not sample for RN-LPN pairs within nursing homes.

Nursing home recruitment was conducted by the PI (AV) via phone calls and onsite visits. Once the administrator and director of nursing agreed to the study, the PI and/or study nurse (AA) went onsite to recruit nurse participants. All eligible nurses were invited to attend face-to-face informational sessions held by the PI (AV) as part of the recruitment process to learn about the study and the researcher’s background in nursing home and medication safety research, and when interested, volunteer to participate.

### Data collection

All interviews were conducted by the PI (AV) or research nurse (AA) in private in either an office or meeting room at the participant’s nursing home. Each interview lasted between 30 to 45 min. Each participant was asked to complete a demographic profile and then to read and respond to questions from four transfer vignettes. Using a previously pilot-tested semi-structured interview guide research staff asked participants to share their perceptions about whether or not a discrepancy was present within each vignette and why. Additional interview questions explored the participants’ experiences with medication reconciliation, the goals of medication reconciliation, and their role with medication safety in the nursing home. The interviews were audio-recorded and transcribed verbatim. Each typed transcript was compared line-by-line with the audio-recorded interviews to assure accuracy. The PI (AV) and research nurse (AA) agreed that data saturation had been achieved at the completion of the interviews, meaning no new data was emerging from participant interviews [19].

### Analysis

We used conventional content analysis [17] in phase one of coding, followed in the second phase by directed content analysis because the study was guided by sensemaking theory [13]. Twenty-six transcripts were loaded into Dedoose® software for analysis. In the conventional analysis phase, initial coding was conducted by three core members of the research team comprised of a PhD-prepared nurse researcher with nursing home and medication safety expertise (AV) and two nursing PhD students (AA, MN). Three transcripts were analyzed jointly to establish consistency and involved the core team reading through the three transcripts together, identifying salient texts, and assigning codes representative of the text. The remaining 23 transcripts were coded independently with each individual transcript discussed among the core team until consensus was reached. Once agreement was reached, codes and related excerpts were placed in a matrix for the next level analysis involving the full research team. The full research team comprised of PhD-prepared experts in nursing home research and sensemaking (RAA), vignette methods (LG), and patient safety (AF), and a doctoral prepared pharmacist with medication reconciliation expertise (LO), reviewed the coded excerpts for validity, making adjustments and collapsing codes where redundancy was evident. This analysis was an iterative process whereby the core team would go back into previously coded data to assure consistency of coding as well as reviewing un-coded texts within each transcript to assure that all relevant text was captured. In the directed content analysis phase, the core team assigned codes and related excerpts to each of the seven sensemaking properties which were then reviewed with the full team to assure credibility. The final level of analysis involved separating RN and LPN coded excerpts and then comparing these data according to the seven sensemaking properties. As with each level of analysis, the final step was conducted by the core team and reviewed by the full team to assure validity.

Three analytical strategies were used to assure findings were credible, dependable, and confirmable [18]. Strategies included: 1) member checking during the interviews to assure a valid reflection of participant perceptions; 2) maintenance of a detailed audit trail to assure data dependability and stability; and 3) participation of expert researchers to assure findings were consistent and objective. These strategies reflect efforts to assure rigor and trustworthiness of the analysis approach.

## Results

A total of 13 RNs and 13 LPNs working in 12 different nursing homes participated in the interviews. One additional RN and four LPNs initially agreed to participate then refused citing work constraints as limiting their time to be interviewed. The 12 nursing homes varied in bed size (42% had 60 to 99 beds, 33% had 100 to 149 beds, and 25% had greater than 150 beds), payer status (50% were for-profit and 50% were not-for-profit), and location (33% were rural and 67% were urban). Among the 13 RNs, 9 had an associate’s degree, 3 had a baccalaureate degree or higher, and 1 had a diploma. All 13 LPNs completed technical training of approximately 12 months. Years of RN experience ranged between 1 year to 41 years; one (8%) had 0–2 years, two (15%) had 3 to 5 years, three (23%) had 6 to 10, four (31%) had 16 to 20 years, two (15%) had 20 to 25 years, and one (8%) had more than 25 years. Years of LPN experience ranged between 1 year to 39 years; two (15%) had 0 to 2 years, one (8%) had 3 to 5 years, five (38%) had 6 to 10 years, two (15%) had 16 to 20 years, and three (23%) had more than 25 years.

Findings are organized according to each of the seven sensemaking properties of identity construction, retrospective, enactment, social, ongoing, extracted cues, and plausibility versus accuracy. A summary of findings associated with each property definition is included in Table 1.

### Identity construction

The sensemaking property of identity construction suggests that members of an organization come to know themselves through interactions with others in the organization [13]. This property was evident when participants spoke about their roles when performing medication reconciliation. RNs mostly spoke about being equal to their LPN colleagues, oftentimes disregarding their advanced RN licensure or higher level of education as contributing to their abilities.

RN: The LPNs [here] have all been nurses longer…I don’t really distinguish our titles…We’re all nurses so that’s how we act, that’s how we treat each other.

RN: I don’t know if it’s because I’m an RN or just because I’m another nurse…I don’t think that [LPNs] look at me and say, “Oh she’s an RN so I can go to her and ask questions.”

RN: So I don’t think [RN or LPN] really differs a whole lot. I think it depends on the types of experience people have had and the jobs they have done before cause a lot of it boils down to experience.

In contrast, while a few LPNs expressed comments such as, “I’ve got a lot to learn” or “I rely on RNs to help me;” LPNs generally perceived themselves equal to RNs in capabilities, skills, and competencies, as evidenced by the following comments:

LPN: the RN is really good with the labs and things. I’m better with orthopedics…the RNs asks me questions…we all, you know, kind of have our strengths.

In a few cases, LPNs characterized themselves as more capable than their RN colleagues exemplified by the following quote:

LPN: We’ve got some dumb RNs, there’s some smarter LPNs, let’s just put it that way. [RNs] question themselves over everything.

LPN: [RNs] might be very book smart but when it comes to being an actual nurse on the floor, a charge nurse, hands on they’re not as good.

These findings suggest that RNs perceive themselves equal to LPNs when performing medication reconciliation despite differences in their education and licensure. In comparison, LPNs also described themselves as equal to and in some cases “smarter” than their RN colleagues, even criticizing the RNs for asking questions about medication orders. This perceived equivalence in skills and abilities on the part of both RNs and LPNs poses a risk to resident safety because collaboration between RNs and LPNs may not routinely occur. Moreover, LPNs criticism of RNs for asking questions demonstrates that LPNs view questioning as a sign of incompetence thus suggesting that LPNs may be less likely to ask questions when clinically necessary.

### Retrospective

The property of retrospective, that individuals make sense of events after they occur [13], was evident when participants shared their experiences about performing medication reconciliation. The majority of RNs shared explicit stories about identifying and resolving medication order discrepancies, as exemplified by the following comments.

RN: I take her blood pressure [on admission] and it runs 90/50 and she returns on five blood pressure medicines…[I] let the doctor know that, do you realize her blood pressure’s running 90/50 and you’ve got her on five different blood pressure medicines.

RN: We had a woman [admitted] who was hospitalized with hyponatremia, muscle weakness and altered mental status…she was on Celebrex [celecoxib] and Celexa [citalopram hydrobromide] and the Celexa is a SSRI [selective serotonin reuptake inhibitor], which can cause hyponatremia, and I have seen this before… the discharge papers said that the Celebrex was DC’d, [sic] and so I questioned it and looked back through the doctor’s notes…I had to call around and get those orders clarified. Anyhow, it was the Celexa that was DC’d [sic] so we, we got it straightened out.

Whereas RNs shared detailed stories, when LPNs were asked about times they identified discrepancies during medication reconciliation, a few shared vague comments such as “I seem to recall a time when…”, or “well, a long time ago…”. However, the majority of LPNs struggled with recalling any examples, stating things such as, “I’m trying to give you examples, [but] I can’t” or “I can’t think of anything.”

The stories shared by RNs of their actual experiences provides evidence that they are, in reality, identifying and resolving discrepancies. These past experiences then provide opportunities to learn, thus influencing the detection of future discrepancies. In contrast, because LPNs are not recognizing discrepancies, they have limited opportunities to learn from past experiences, and future discrepancies may go undetected and unresolved.

### Enactment

Enactment is when individuals mentally create what they expect to find [13]. Enactment was evident in participants’ responses about why they performed medication reconciliation and what discrepancies they perceived were present/not present within the vignettes and why. RNs described performing medication reconciliation primarily to “limit medication errors” because they anticipated discrepancies would occur, therefore order clarification would be necessary. RNs also spoke about the resident’s clinical history and the need to know about his/her hospital stay to better understand what medications were ordered and why so they could identify if an intentional order change versus an error had occurred.

RN: [This resident was] taking Glucophage (metformin) at home prior to hospitalization and at the hospital and then it’s like it got dropped out with the transferring information…I would question that order change.

RN: [Resident] became acutely ill with pneumonia, already has a diagnosis of heart failure, so when she was in the hospital they bumped her up to [Lasix (furosemide)] 80mg, which indicates to me that her heart failure was coming into play somehow, and then when they transferred her back [to the nursing home] they dropped her back down to the 40mg, which it, it’s a discrepancy because I want to know why they dropped her back down.

The majority of LPNs stated that medication reconciliation was about matching medication orders between settings, stating, “We go through and we check every single [order] against each other.” However, unlike RNs who anticipated order changes would occur and sought to clarify differences, most LPNs described transfer orders as typically written as intended, then proceeded to make assumptions about why medication order changes may have occurred.

LPN: And [I] just assuming that she didn’t need [Glucophage (metformin)] anymore …because I’m sure her blood sugars were running fine.

LPN: I’m assuming they increased her Lasix (furosemide) while she was in the hospital, probably because she was having some fluid issues… then they got that resolved and backed her [dose] back down.

These findings suggest that RNs more often anticipated medication order changes occurred at transfer, which underscored their rationale for clarifying when an intentional change versus an error may have occurred. LPNs, on the other hand, more often anticipated orders were written as intended, then made assumptions about why order changes occurred, despite not having any clinical information in the vignette to support their assumptions. Making assumptions about any medication order change can result in potentially harmful errors going undetected and unresolved.

### Social

The sensemaking property of social proposes that individuals are influenced by relationships and interactions with others in the organization [13]. This was evident when participants spoke about seeking information when performing medication reconciliation. RNs described adversarial relationships with physicians and hospital nurses, specifically when questioning medication orders changes to identify errors.

RN: So I think fear of reprisal from the doctor sometimes really diminishes nurses’ ability to speak up and say “Hey, can you explain this to me, or why is it this way,”

RN: I got a little bit of flak about checking on the discrepancy. The [hospital nurse] was questioning why we were questioning the order. They don’t like you to question orders.

LPNs lack of experience with identifying discrepancies hindered their ability to talk about resolving discrepancies. However, when discussing their overall roles in the nursing home to assure medication safety, LPNs often shared examples of positive relationships with others, including physicians.

LPN: Most [physicians] all trust me…they’ll tell me to go look at something and then just write it, you know, write it for whatever you think and so, yeah, that’s good to have that kind of rapport.

LPN: Well, I do [help others], especially the [name omitted, unit LPN] who hasn’t been out of school that long, she does call me with questions all the time.

These findings suggest that RNs encounter resistance when questioning medication orders; perhaps because RNs are more likely to question order accuracy. LPNs’ stories about how others trust their judgment may reflect a desire to be viewed positively by others. Overall, the LPNs’ lack of experience with identifying medication order discrepancies limits our understanding of how they might interact with others to resolve discrepancies. Moreover, the resistance described by RNs might actually inhibit any nursing home nurse, including both RNs and LPNs from questioning medication orders that require clarification.

### Ongoing

The property of ongoing submits that an event’s history and context influence an individual’s ongoing understanding of future events [13] and was evident when participants spoke about discrepancies within the context of how they performed medication reconciliation. RNs spoke more often about medication safety by focusing on medication type, including both high-risk and low-risk medications.

RN: I mean, anticoagulants, any kind of narcotics, anything to do with CHF [congestive heart failure], diabetic medications…we’re not that concerned about vitamins,

RN: You know, it’s [PRN laxative] kind of an innocuous medication, but it is still something different, I would ask, …is he having trouble, then when I call the doctor say, “Hey [the hospital] added this because, you want to continue it?”

LPNs, on the other hand, spoke less about medication type and more about what they assumed would “typically” occur with medication orders as part of a resident transfer.

LPN: Everybody comes back [from hospital] on Colace (docusate) basically because of the inactivity and a lot of times they’re on pain medications and constipation is a problem. I wouldn’t question Colace (docusate) in the least.

LPN: [Resident] had a urinary tract infection. She’s 84 years old. Typically they are taking calcium. Why would she need more calcium? It was a urinary tract infection…. [But] It’s not gonna hurt to take it.

RNs further commented that changes in medication orders, even when intended, might require clarification, particularly if the resident could be adversely affected.

RN: [Resident] had pneumonia and something else, and they took him off all of his psych [sic] medications while he was at the hospital. He is extremely schizophrenic and those are not things that you can abruptly stop.

RN: [Resident came] back recently from the hospital that was here before and did not come back on their antidepressant. That was a big one because that was something that we had addressed while they were here…the hospitalists don’t know these people from Adam, and a lot of times we won’t get back the orders that maybe they left on that were important, like the antidepressant.

These findings suggest that RNs consider medication reconciliation in the context of resident safety, including a focus on both high-risk and low-risk medications, taking into account the resident’s clinical history and hospital events to assure medication appropriateness. LPNs, on the other hand, considered medication reconciliation and what they anticipated should occur within the routine transfer process. Once again, this provides evidence that potentially harmful discrepancies can go undetected and unresolved.

### Extracted cues

The property of extracted cues suggests that individuals will extract cues out of familiar structures or known points of reference, such as rules, within their environment [13]. Extracted cues were evident when participants referenced what guided their thinking about what discrepancies might exist. Both RNs and LPNs also talked about nursing home rules and regulations as guiding their thinking. For example, when antipsychotic medications were ordered, both RN and LPN participants raised questions because, as one RN stated, these medications are “always a red flag” in nursing homes. RNs more often sought to understand the clinical reason for the antipsychotic order, however, whereas LPNs were simply focused on the regulation and the need to “do away” with the order.

RN: And I would question several sources for that [order] because I don’t like giving an antipsychotic if I don’t have to, I would question whether or not [staff] were trying to control some behaviors in the hospital.

LPN: Because of the way the [regulators]…are now really trying to work away, getting away from antipsychotics. Okay? There’s a big push for nursing homes to do away with them.

Only RNs, however, shared examples of clinical reasoning. In particular, RNs spoke about medication effects and their knowledge of clinical conditions to further explain how they identify discrepancies.

RN: [Hospital] had almost doubled the Lasix that the [resident] was taking and had also increased the Prednisone…and I questioned that because it seemed like it was rather a large [change] all at one time that it, without titrating.

RN: [Resident] actually had bronchitis, okay, which yeah, turned to pneumonia, and it’s like yeah, okay, I see the reason for the steroids [during hospitalization]…I understand [steorids] makes the blood sugar jump… [but] why is she continued on insulin [at transfer] because there is no diabetic history.

These findings suggest that both RNs and LPNs reference nursing home rules/regulations as cues to identify discrepancies. However, RNs were more likely to engage in clinical reasoning by sharing examples of their expanded knowledge of medication therapy and clinical practice to inform how they identify discrepancies. Perhaps these examples of clinical reasoning provide evidence that RNs have a richer mental model from which to identify discrepancies and may be a result of their higher level education.

### Plausibility rather than accuracy

Plausibility rather than accuracy suggests that individuals seek to behave in a reasonable fashion within the context of the unexpected event so they can move quickly past it [13]. This property was evident when participants spoke about the actual process of medication reconciliation. Both RNs and LPNs talked about the complexity of the process, particularly about time constraints and competing demands as sources of frustration.

RN: Not having time to actually totally focus on what I’m doing without being drawn away, especially on an admission.

LPN: When you get all the paperwork from the hospital…and you have to go through and read because they don’t always put every order that they want.

LPNs further acknowledged that errors inevitably occurred while trying to get the process done.

LPN: You can’t always be completely a hundred percent confident unless you were to call back and verify every single order because maybe it could still be an error…and that [call back to verify] won’t happen.

LPN: We get errors sometimes and the way some nurses, you know, will take off an order…I’ve made med [sic] errors before…

RNs shared concerns that even though their LPN colleagues are “faster,” they questioned the accuracy of their work, stating, “[LPNs] are faster…but it is correct, it is right?” One RN shared concerns about LPNs and how she approaches medication reconciliation differently.

RN: I don’t want to sound RN snobby, but I have seen LPNs just write down whatever is on the transfer sheet and, and not really think about it. I think about it, you know, think about what this person’s taking this drug for, what is this person’s diagnosis…you have to look at the whole picture…LPNs get kind of focused and don’t look at the whole thing.

These findings suggest that both RNs and LPNs consider their approaches to medication reconciliation within the constraints of time and competing demands. However, RNs acknowledged medication reconciliation was a complex cognitive process and that despite competing demands, being faster rather than being comprehensive compromised accuracy and safety.

## Discussion

Findings from this study confirm our earlier work that nursing home nurses are in a unique position to identify medication order discrepancies, particularly at resident transfer, because of their frontline proximity to residents, families, and providers at the time of transfer [11]. However, this study adds new knowledge about how RNs and LPNs actually differ in practice when identifying discrepancies, an important finding since RNs and LPNs are often used interchangeably in nursing homes in clinical decision-making roles such as medication reconciliation [11, 12, 17].

RNs clearly contribute to positive resident outcomes [20–22], including identifying potentially harmful high risk medication order discrepancies [17], yet little is known about how RNs actually differ from LPNs in practice to influence outcomes. Findings from this study provide important insight into these differences specific to medication reconciliation by suggesting that RNs more often engage in effective sensemaking behaviors when identifying discrepancies. Effective sensemaking, believed to facilitate actions that promote safety rather than hinder it, occurs when individuals cognitively challenge assumptions, question or raise doubt, seek others input, and openly share information. In contrast, behaviors that inhibit effective sensemaking occur when individuals prefer formal processes (rules), reject complex explanations, make assumptions, and push for conclusions [23]. In this study, RNs were more likely to challenge assumptions about medication orders, raise clinical questions about medications in relation to the residents’ medical history, learn from past experiences, and seek others input to guide their decisions. LPNs on the other hand were more likely to make assumptions about medication orders, to not recognize the complexity of resident conditions, and more often rely on rules to guide decisions. RNs may be more effective at sensemaking because of their expanded education beyond that of LPNs, therefore they may recognize and respond to clinical cues differently thus guiding safer actions.

Regarding the process of medication reconciliation, whereas LPNs were focused on the task, RNs were focused on preventing harm. Not only were RNs alert to potential errors, they also questioned medications that may have been intentionally ordered at transfer yet posed risk for resident harm. Questioning medication orders that may have been prescribed intentionally can still pose risk [4] and is another aspect of medication reconciliation critical to preventing potential harm [7, 8]. When probed about their personal experiences, RNs acknowledged that their LPNs colleagues were often “faster” when performing medication reconciliation which may be desired in the rapid paced nursing home environment, particularly among nursing home leaders. Yet, RNs also described concerns about the accuracy of LPNs’ work suggesting the risk of moving through tasks quickly leaves residents vulnerable to error and potential harm.

Despite acknowledged differences between RN and LPN approaches to medication reconciliation, RNs generally perceived themselves to be comparable to their LPN colleagues in overall skills and abilities. In contrast, LPNs expressed a belief that they are equal to the RN or in some cases, superior. These disparate perceptions may result from the interchangeability between RN and LPN assigned responsibilities in the nursing home, in which organizational leaders may not value the RN contribution as unique from that of the LPN. Additionally, LPNs’ overconfidence in their abilities may in reality be driven by fear and frustration of being viewed incompetent because of organizational expectations to practice beyond their knowledge and skill [24]. Moreover, the LPNs overconfidence and/or their fear of being perceived as incompetent will likely influence their unwillingness to collaborate with the RN about matters related to medication safety thus further contributing to potential resident harm.

In the U.S., RN and LPN levels of education and scopes of practice differ [16]. The findings from this study demonstrated the power of education in shaping mental models for sensemaking. Education for RNs includes 2 to 4 years within an accredited nursing program and are licensed to provide autonomous comprehensive nursing care including assessment, care planning, delegation and supervision. In comparison, LPNs attend a 12-month program and have a limited, more technical scope of practice. In nursing homes however, this study and other studies provide evidence that RNs and LPNs are treated as interchangeable in their contribution to resident care [11, 25–28]. The quotes from the RNs and LPNS in our study support this drive to perceived equivalence in their roles. Despite this strong influence of identify construction of nurses in nursing homes, the fundamental difference in professional education and socialization had an impact in shaping the mental models of RNs and LPNs. The RNs were more capable of complex, critical thinking and approached the task of reconciliation skeptically. LPNs on the other hand demonstrated less ability for critical thinking, relied on assumptions, and in some instances seemed over confident in their ability, which is an attitude associated with errors [23].

There are important practice implications for improving medication reconciliation processes in nursing homes. First, medication reconciliation in nursing homes requires moving beyond a checklist approach. Comparing medication orders between settings is a part of the process [7, 8], however, a checklist alone is not enough because it does not guide nurses, particularly LPNs to question orders including orders intentionally changed that could cause harm. Medication reconciliation requires a collaborative approach that moves beyond the checklist, where medication orders are questioned within the context of the resident’s clinical condition and then clarified with providers to assure appropriateness. The cognitive nature of medication reconciliation [7, 8] should require RNs be assigned to oversee the process in nursing homes, working collaboratively with other staff including LPNs, residents/families, providers, and pharmacists Although study participants, particularly RNs, often mentioned collaborating with physicians, they did not explicitly mention pharmacists, perhaps because pharmacists are offsite from the nursing home at the time of resident admission [11, 12]. Pharmacists however are integral to medication reconciliation [10] and are a valuable resource for nursing home staff. Future research should focus on designing medication reconciliation processes that move beyond the checklist to a structured comprehensive and collaborative process that directs staff to anticipate potential harm.

### Limitations

We acknowledge a number of limitations within this study. First, although the nursing home sites were randomly selected, participants within each site were selected based on self-report that they participated in medication reconciliation at their nursing home. Second, because medication reconciliation practices vary across settings, it is possible processes within these nursing homes are unique. Third, vignettes represent hypothetical situations, therefore, they can vary somewhat from practice. To minimize this limitation, we developed each vignette from our previous observational work of actual resident transfers therefore reflecting medication order discrepancies that nursing home RNs and LPNs encountered. Also, this study did not take into account nursing home factors at the organization level; therefore, factors such as RN and LPN assigned roles, leadership style, and the safety culture could influence how nurses detect medication order discrepancies and should be considered in future studies.

## Conclusions

Adverse drug events resulting from medication order discrepancies pose a real risk to resident safety. Nursing home nurses, particularly RNs, are in an important position to identify discrepancies that could cause resident harm. Both RNs and LPNs are valuable asset to nursing home care and keeping residents safe, yet RNs offer a unique contribution to complex processes such as medication reconciliation. Nursing home leaders must acknowledge the differences in RN and LPN contributions and make certain the most qualified practitioner is assigned to ensure residents remain safe.

## Abbreviations

AHRQ: Agency for Healthcare Research and Quality
LPN: Licensed Practical Nurse
RN: Registered Nurse
TJC: The Joint Commission
